# Supplementary material for: The Toxoplasma gondii F-Box Protein L2 Functions as a Repressor of Stage Specific Gene Expression
Source: PLoS Pathog. 2024 May 30;20(5):e1012269. doi: 10.1371/journal.ppat.1012269 (PMC11166348; doi:10.1371/journal.ppat.1012269)

Figure S1. TGME49_313200, 10 exons, 832 aa

A. Predicted domain organization after HA-tagging. LRR = leucine-rich repeat, NLS = monopartite nuclear localization sequence. LRR3 and LRR4 are predicted by alphafold (see panel D) but are not identified by sequence prediction algorithms. Regions not labeled are poorly conserved.

B. Predicted amino acid sequence (from Toxo.db). Predicted domains are labeled and numbered as indicated. Underlined sequences were confirmed by mass spectrometry. Double underlined sequence represents the N-terminal 3X HA-tag.

C. Organization of amino acids 305-832 as predicted by alphaFold-2 (<https://alphafold.ebi.ac.uk/entry/S8EUA4>).

A.

`

1 MYPYDVPDYAGYPYDVPDYAYPYDVPDYAMLEARNASRADAARGPLRGTQGDEQTAERKE

61 TERARAQENADTFNTRSAKMSETVVNMREKEGREKGEQKEKKGQVAEEKKERRSERERPK

121 EKDLKSLFDSTDSDCLTSLETAKRALPKVEKKQMTILSFFSRPSSSSASSSPPSSSPPSS

NLS1

181 FSSSSSASSPSLLFRVPSALSRAASSTHTTEKRPTGPVDEKEEKKLLKRRRVCASEETDA

NLS2

241 LQGCARSFLAGMREAPRAGSASEANSKGEMSRDANVDSLHTPRLVEEHRKRRRTRLIQEH

F-box

301 RAHNSSSSSSSSSSSSSSSSSSSSSSEKIELSPVGLSDLPEELLQQILDCCPKECLLVSH

F-box LRR1 LRR2

361 ALAAAVRRRRRVLRLTPLQCTEPRAEQFLSAIRSYPQLRLLEIVNAPWLSARFIENWANS

LRR2 LRR3 LRR4

421 KSSYFPQRLEVFVLRKCPRLTSRLARVLTTRLRRLRAIDLQDNSSLDYTSVVYLRTLPFL LRR4 LRR5.1

481 ERVALGVSPSNTRGTSAHCNLTLCALLGPPSSSPTLLPSSTSSVLSSSSSSSLACSSASS

541 SVCPSTSTSPPSCLPSPNSTPVSSSSSSPRSPPSSLRWSGEKGEGREGEENEKRGSGSAL

LRR5.2 LRR6

601 PRSKDEAGAGQAVREQRGETRAVDLLEKAQKQKQDQTAGSSSPPLKLLSLARCAALSSLA

LRR6 LRR7

661 PLINVASTLEFLDLRGCCALDDSSAAVLASLANLQVLVLSDTGVTSTTVAAVAENCRRLE LRR8 LRR9 LRR10

721 MLDVSRISRFSKEAALLLPLHLLRLTRLKLTKNSAVDDEVVRDCLSRLKRLALLDVSHCW LRR11 LRR12

781 RVTSGFCVPPLPQNPDGMSLRRLGLFGCNVERQRVQDALRDAGAAKVQLSLHHELPMFDL

841 PCVYSNLPNLDLRCRTFIEGC*

D. Predicted organization of amino acids 305-832 (alphaFold-2). The F-box-like region is in red, and the 12 predicted LRRs are in cyan. Note that LRR5 is split by a long loop, not shown.


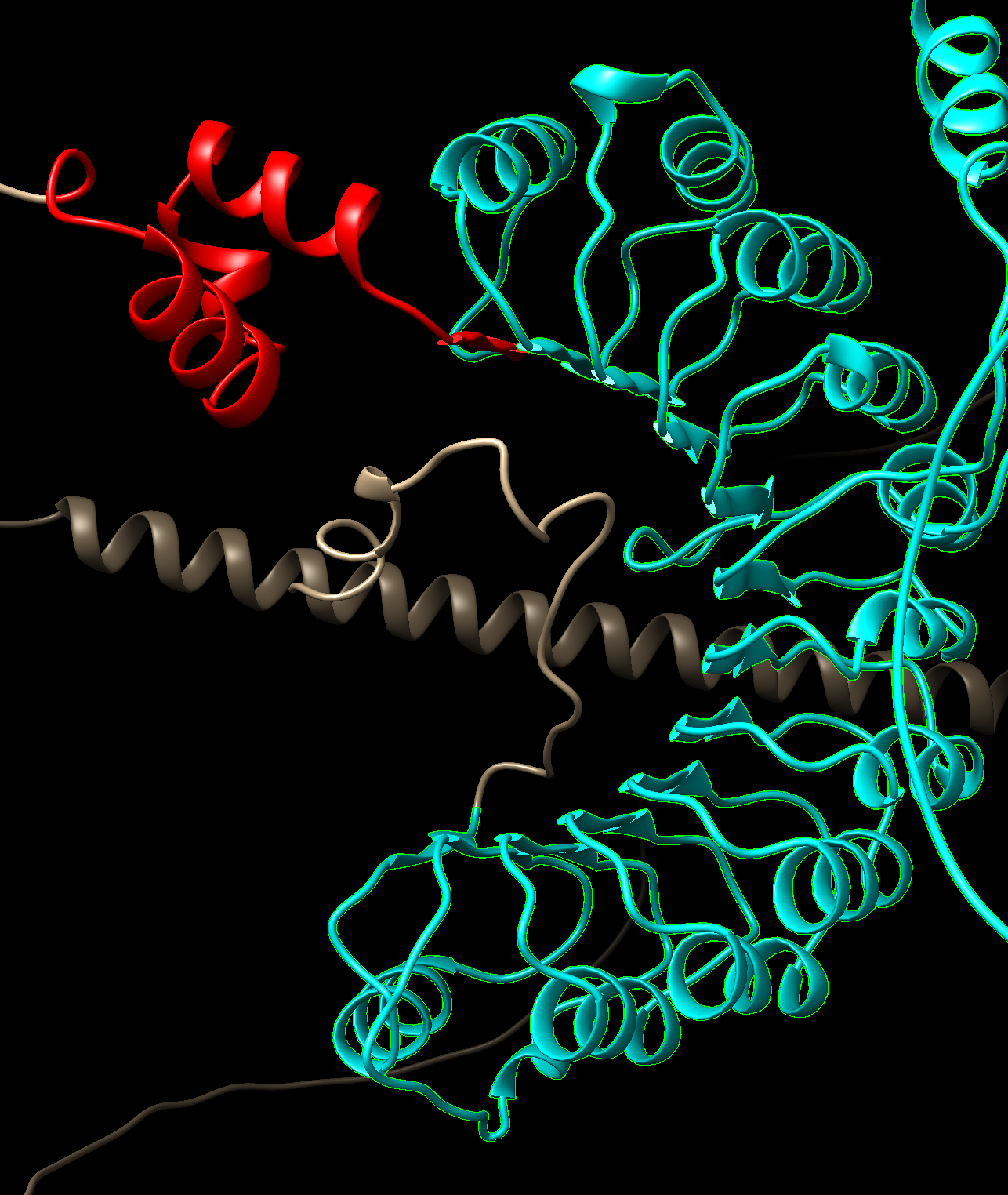

Supplement: S1 Fig — (A). Predicted domain organization after HA-tagging. LRR = leucine-rich repeat, NLS = monopartite nuclear localization sequence. LRR3 and LRR4 are predicted by alphafold (see panel D) but are not identified by sequence prediction algorithms. Regions not labeled are poorly conserved. (B). Predicted amino acid sequence (from Toxo.db). Predicted domains are labeled and numbered as indicated. Underlined sequences were confirmed by mass spectrometry. Double underlined sequence represents the N-terminal 3X HA-tag. (C). Organization of amino acids 305–832 as predicted by alphaFold-2 (https://alphafold.ebi.ac.uk/entry/S8EUA4). (DOCX) [file ppat.1012269.s001.docx]
