# Supplementary figures and images for: The Toxoplasma gondii F-Box Protein L2 Functions as a Repressor of Stage Specific Gene Expression
Source: PLoS Pathog. 2024 May 30;20(5):e1012269. doi: 10.1371/journal.ppat.1012269 (PMC11166348; doi:10.1371/journal.ppat.1012269)

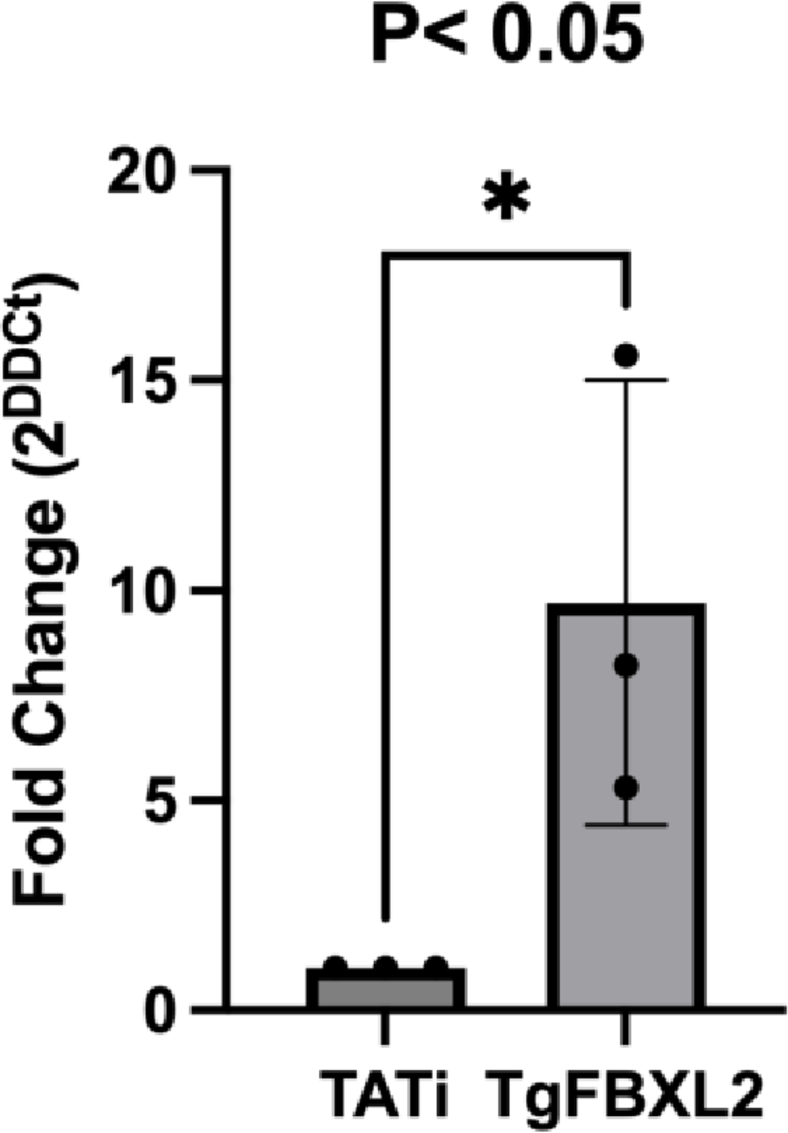

Supplement: S2 Fig — qPCR was used to quantify TgFBXL2 mRNA levels in HATgFBXL2 parasites and parental TATiΔKu80 strain. Parasites were grown for 24 on HFF monolayers. Actinonin and clindamycin were used as positive inhibitors of apicoplast genome replication. Shown are means and standard deviations from 3 independent experiments, normalized by actin. (P <0.05, one-way ANOVA). (TIF) [file ppat.1012269.s002.tif]

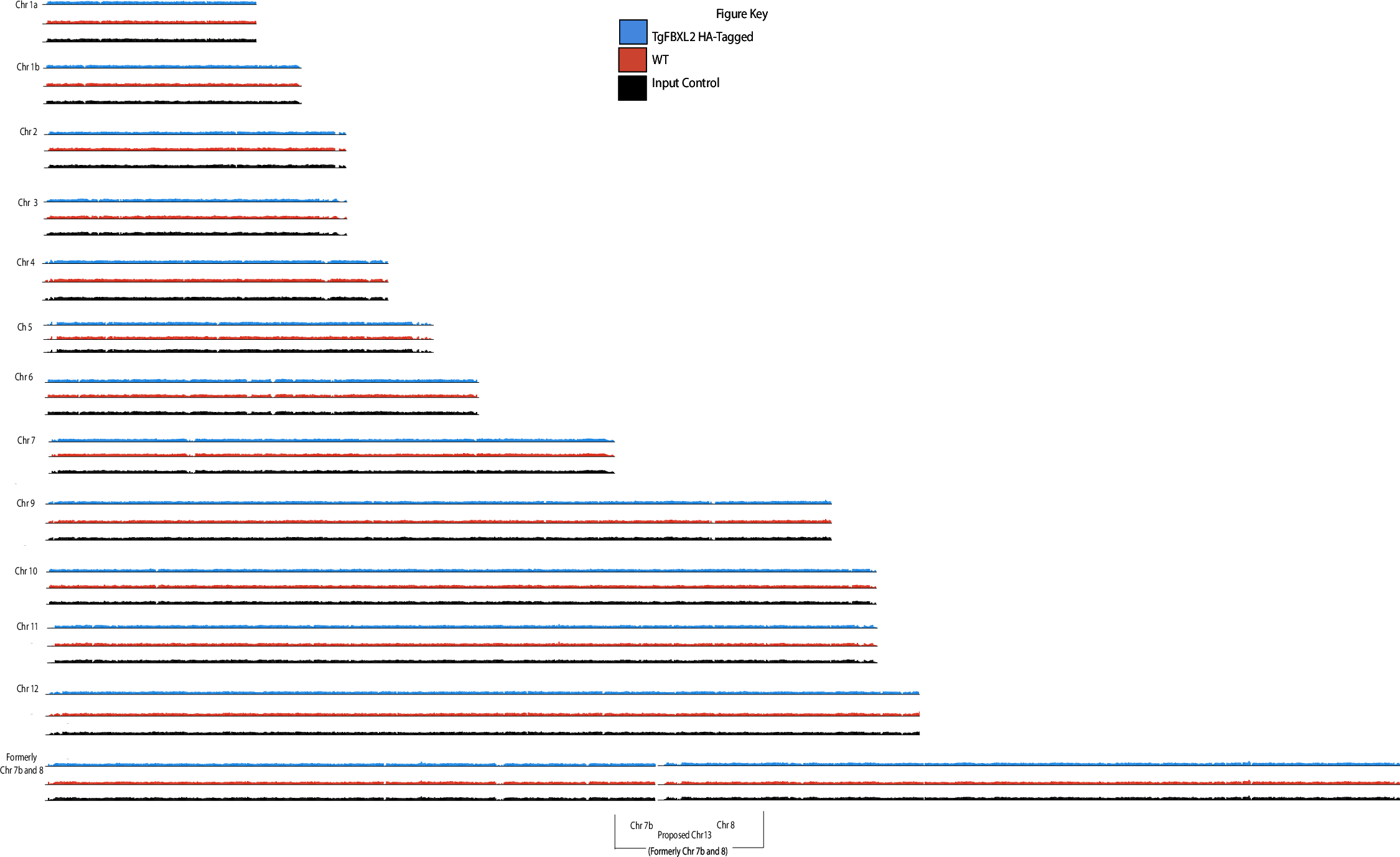

Supplement: S3 Fig — ChIPSeq results visualization of HA tagged TgFBXL2 parasite line (TgFBXL2-HA) across all 13 of Toxoplasma’s chromosomes. Reads were normalized to millions of mapped reads. Tracks correspond to TgFBXL2-HA tagged parasites in blue, wild type parasites in red, and an input control in black. Former chromosomes 7b and 8 were combined into the recently suggested chromosome 13 [88,89]. (TIF) [file ppat.1012269.s003.tif]

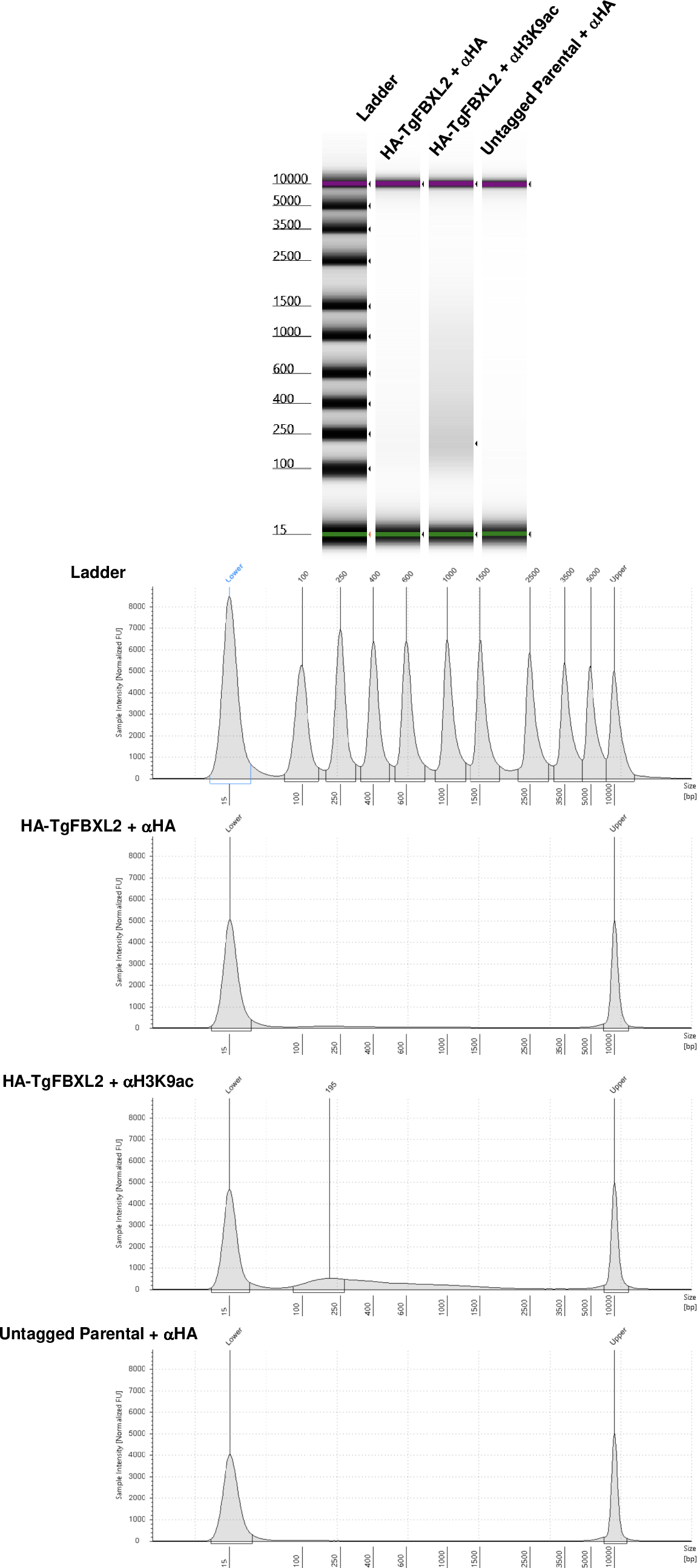

Supplement: S4 Fig — Tapestation analysis of DNA purified from HATgFBXL2 ChIP. Gel image representing three ChIP DNA samples from HATgFBXL2 immunoprecipitated with anti-HA, HATgFBXL2 immunoprecipitated with anti-H3K9ac (positive control) and the parental TATi parasites immunoprecipitated with anti-HA. Electropherograms from each sample depict marker peaks at 15 and 10,000 bp, with a peak of immunoprecipitated DNA at 195 bp present only in the positive control. (TIF) [file ppat.1012269.s004.tif]

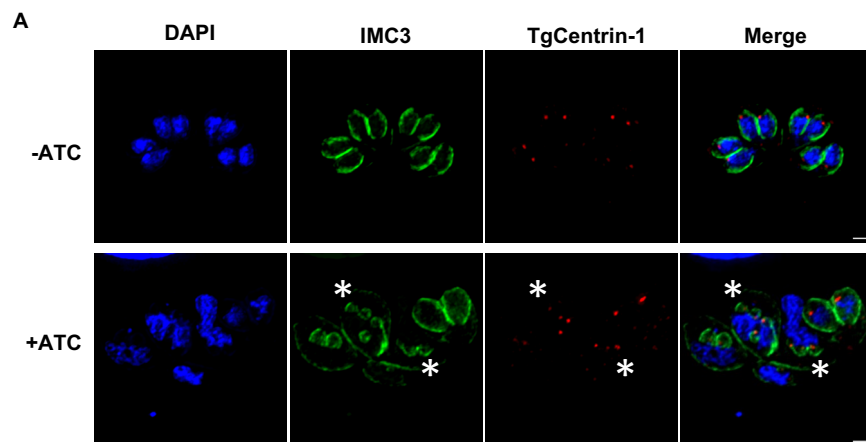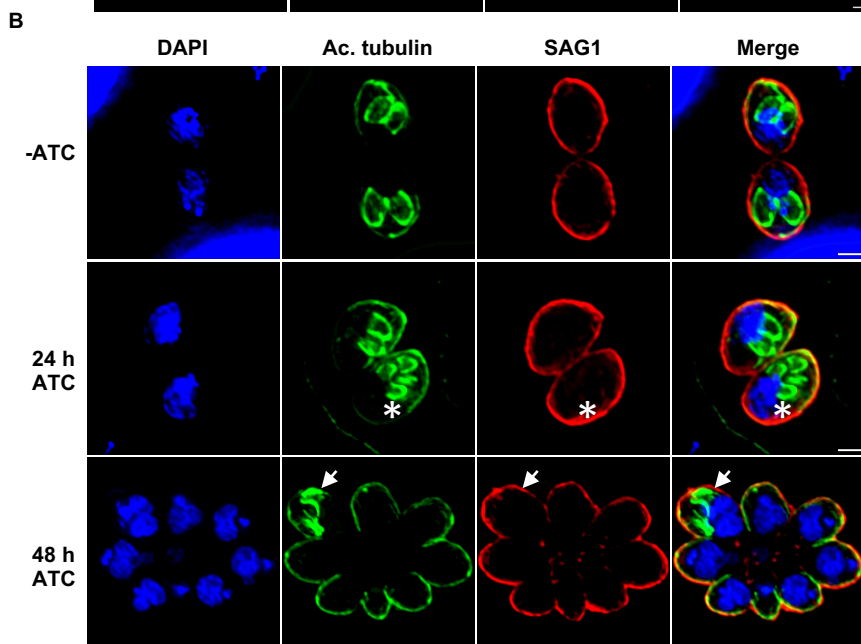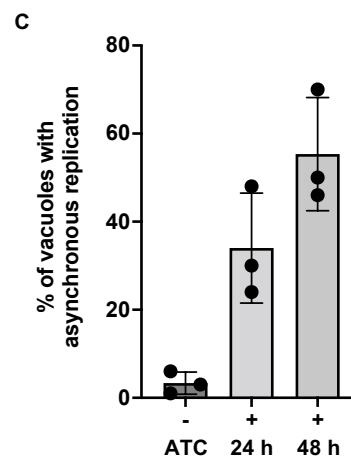

**Fig. 3**



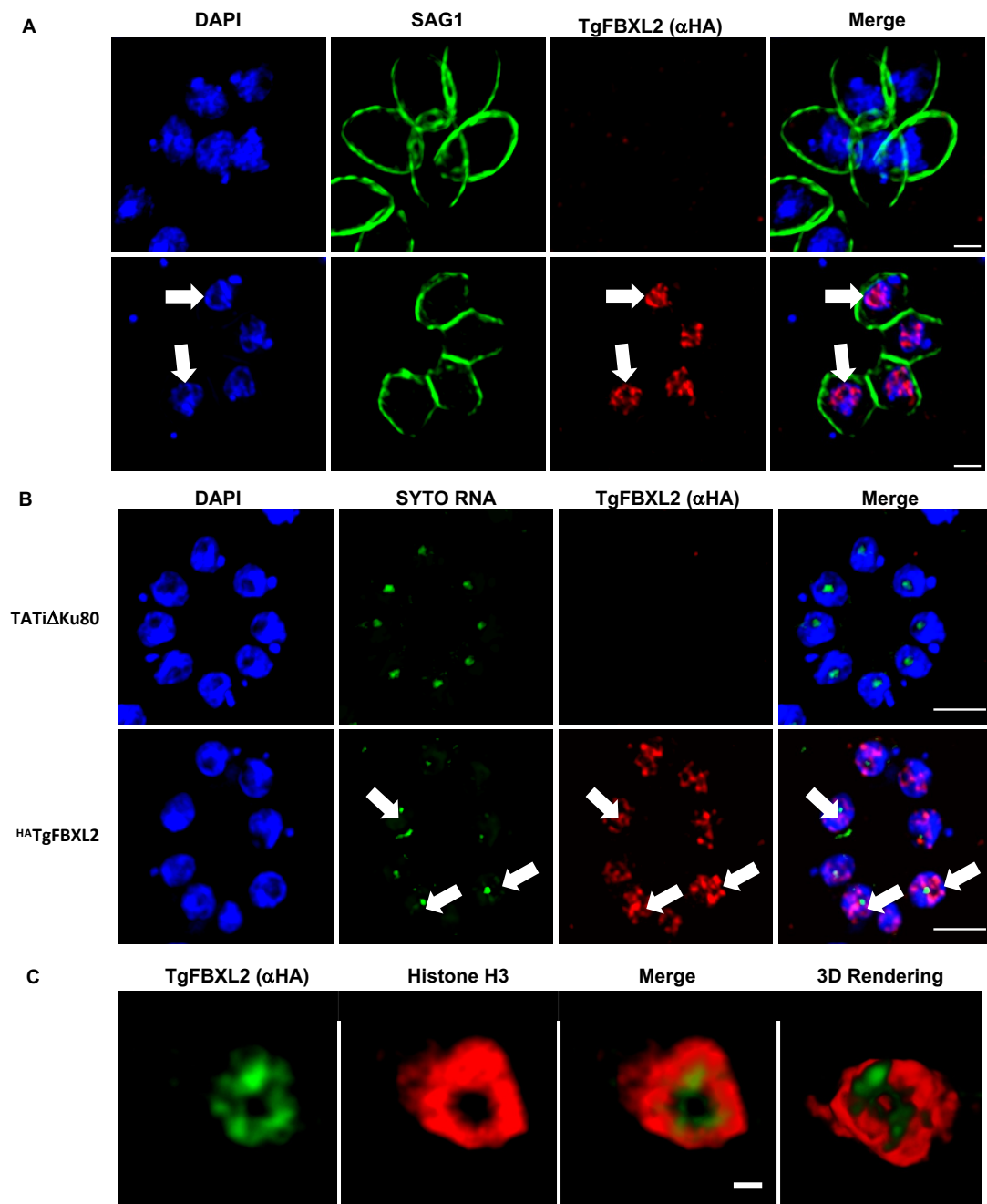

**Fig. 5**

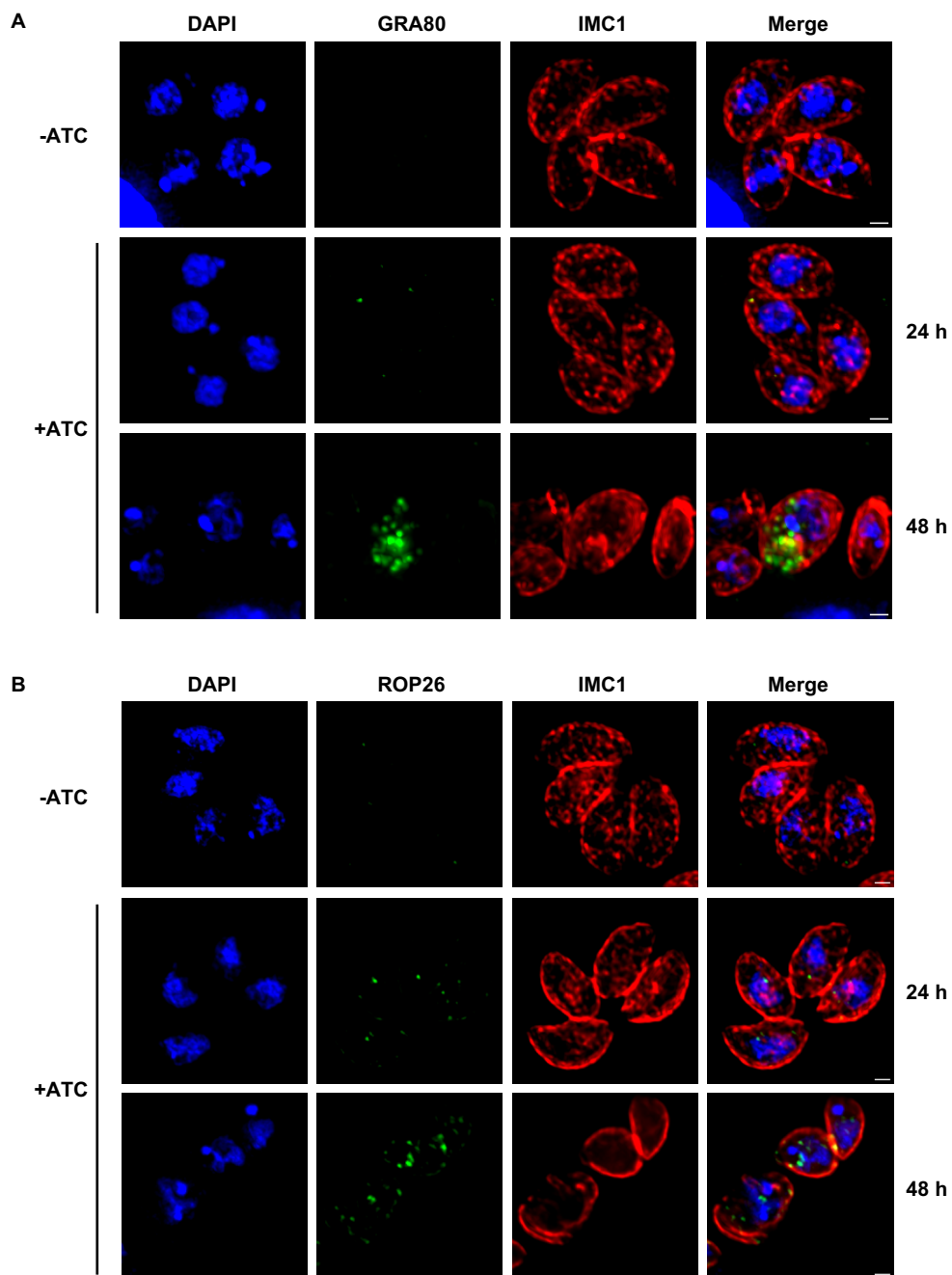

**Fig. 7**

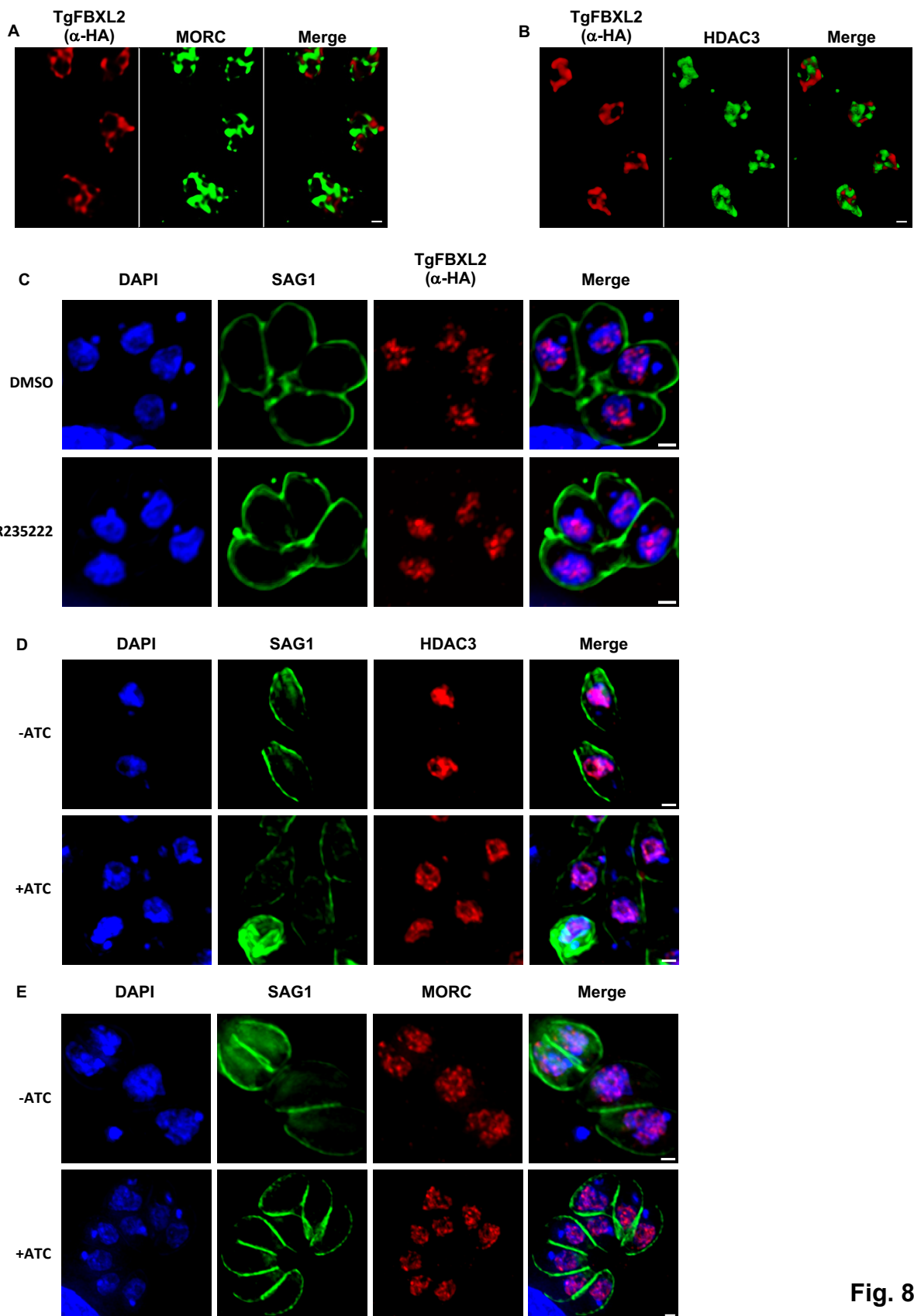

**Fig. 8**

Supplement: S5 Fig — (PDF) [file ppat.1012269.s005.pdf]
